# Myocardial feature tracking for viability assessment in ischemic cardiomyopathy

**DOI:** 10.1186/1532-429X-13-S1-P153

**Published:** 2011-02-02

**Authors:** Andreas Schuster, Matthias Paul, Nuno Bettencourt, Tarique Hussain, Dirk Lossnitzer, Masaki Ishida, Amedeo Chiribiri, Boris Bigalke, Geraint Morton, Divaka Perera, Gerald Greil, Eike Nagel

**Affiliations:** 1 King's College London BHF Centre of Excellence, Division of Imaging Sciences, NIHR Biomedical Research Centre at Guy's and St. Thomas' NHS Trust Foundation, Wellcome Trust and EPSRC Medical Engineering Centre; The Rayne Institute, St. Thomas´ Hospital, London, UK; 2 Cardiology Department - Centro Hospitalar de Gaia/Espinho, Porto, Portugal

## Objective

To evaluate myocardial feature tracking (FT) as a potential novel quantitative analysis tool for the assessment of myocardial viability using LDDSMR.

## Background

Low dose dobutamine stress magnetic resonance imaging (LDDSMR) is a widely accepted technique for assessment of hibernating myocardium in patients with ischemic cardiomyopathy (ICMP). It is particularly helpful in patients with intermediate transmurality of scar where prediction of functional recovery by late gadolinium enhancement imaging (LGE) alone is difficult. Analysis of LDDSMR is usually based on visual assessment and is therefore considerably operator dependant. Recently myocardial feature tracking (FT) has been introduced. It tracks tissue voxel motion of cine-MR images to assess circumferential and radial myocardial strain independent of additional sequences.

## Methods

15 consecutive patients with ICMP referred for viability assessment were studied at 3 Tesla (Philips Achieva) at rest and during LDDSMR. ICMP was defined as: angiographically established coronary artery disease, LV-EF ≤45% and ≥2 segments with wall motion abnormalities at rest. Myocardial function was studied by steady state free precession (SSFP) cine imaging in 3 short axis slices covering 16 myocardial segments, excluding the apex.

The same segments were studied with dedicated FT software (Diogenes MRI, Tomtec, Germany) to assess subendocardial and subepicardial circumferential (Ecc) and radial (Err) strain.

## Results

208 of 240 segments (87%) were analysed by two independent observers. 32 segments were excluded because of insufficient image quality or visualization of the outflow tract. Interobser-variability was: Ecc _subendocardial_ r=0.84, Ecc _subepicardial_ r=0.72 and Err r=0.7.

During LDDSMR (5 and 10 µg · kg^-1^· min^-1^) there was improvement in Ecc _subendocardial_, Ecc _subepicardial_ (p<0.001, by repeated measures analysis) and Err (p=0.001, please refer also to Table 1). Normokinetic segments without scar (n=67) improved in Ecc _subendocardial_ (p<0.001) and Ecc _subepicardial_ (p=0.008) while Err remained unchanged with stress. Hypokinetic segments without scar (n=75) improved in all three strain values (Ecc _subendocardial_ p=0.012 ; Ecc _subepicardial_ p=0.035 ; Err p=0.001). Hypokinetic segments with non-transmural scar (n=60) showed improved Ecc _subendocardial_ , Ecc _subepicardial_ and Err up to 75% transmurality while transmurally scarred segments (n=6) remained unchanged with stress.

**Table 1:** Strain Values for different groups of transmurality at rest, 5 and 10 µg · kg-1· min-1 of dobutamine.

Strain	Strain Rest, 5 and 10 µg · kg^-1^· min^-1^ of dobutamine	p
All segments (WMS 0-5, LGE 0-100%, n=208)		
Ecc _subendocardial_	-11.8±8.9 ; -14.1±9.7 ; -16.2±11.2	**<0.001**
Ecc _subepicardial_	-7.1±5.2 ; -8.9 ±6.1 ; -10.4±7.8	**<0.001**
Err	13.4±12 ; 16.1±12.1 ; 17.2±14.3	**0.001**
Normal segments (WMS 5, LGE 0%, n=67)		
Ecc _subendocardial_	-16.2±11.1 ; -18.9±11.5 ; -22.3±13.3	**<0.001**
Ecc _subepicardial_	-8.6±6.2 ; -10.6±7.4 ; -13±10.3	**0.008**
Err	19.3±15 ; 20.7±13.2 ; 21.4±15.7	0.675
Hypokinetic segments without scar (WMS<5, LGE 0%, n=75)		
Ecc _subendocardial_	-10.5±6.9 ; -12.1±6.9 ; -14.1±9.2	**0.012**
Ecc _subepicardial_	-7±4.8 ; -8.2±5.5 ; -9.1±5.9	**0.035**
Err	11.7±8.3 ; 16±10.9 ; 16.5±12.8	**0.001**
Hypokinetic segments with non-transmural scar (WMS<5, LGE 1-25%, n=16)		
Ecc _subendocardial_	-10.7±7.9 ; -16±8.7 ; -16.6±10.8	**<0.001**
Ecc _subepicardial_	-7.6±5.3 ; -10.4±6.6 ; -12.2±7.5	**0.016**
Err	11.7±7.19 ; 20.1±16.8, 20.3±18.6	0.075
Hypokinetic segments with non-transmural scar (WMS<5, LGE 26-75%, n=44)		
Ecc _subendocardial_	-7.6±5.2 ; -10.3±7.4 ; -11.8±7.7	**<0.001**
Ecc _subepicardial_	-5.6±3.8 ; -7.4±4.3 ; -8.5 ± 4.9	**0.001**
Err	8.4±10.1 ; 8.7±5.6 ; 12.3±12	**0.017**
Hypokinetic segments with transmural scar (WMS<5, LGE 76-100%, n=6)		
Ecc _subendocardial_	-4.7±3 ; -2.9±2.5 ; -6.6±3.3	0.214
Ecc _subepicardial_	-2.9±2.9 ; -5.4±3.9 ; -4.5±4.2	0.131
Err	9.51±5 ; 5.4±6.2 ; 4.9±3.3	0.543

## Conclusion

FT is a novel technique, which reliably detects quantitative wall motion derived from SSFP CINE imaging. It seems useful for quantitative analysis at rest and during low dose dobutamine stress for viability assessment in patients with ischemic cardiomyopathy.

